# The mechanism study of miR‐125b in occurrence and progression of multiple myeloma

**DOI:** 10.1002/cam4.1181

**Published:** 2017-12-07

**Authors:** Da Gao, Zhen Xiao, Hui‐Ping Li, Dong‐Hai Han, Ya‐Peng Zhang

**Affiliations:** ^1^ Department of Hematology the Affiliated Hospital of Inner Mongolia Medical University Hohhot 010050 China

**Keywords:** lncRNA MALAT1, MiR‐125b, multiple myeloma, notch‐1 pathway

## Abstract

Although many efforts have contributed to improve our knowledge of molecular pathogenesis about multiple myeloma (MM), the role and significance of microRNAs and long noncoding RNAs in MM cells, along with the core mechanism remains virtually absent. The mRNA levels of miR‐125b and MALAT1 in MM cell lines were detected by qRT‐PCR. The influence of Lenti‐Sh‐miR‐125b on cell viability and the Notch‐1 pathway‐related proteins were assessed by MTT method and western blot, respectively. We also investigated the regulation effect between MALAT1 and Notch1 pathway. Moreover, the connection between Notch1 signaling and MM cell growth was discussed in‐depth. The reverse effect of pcDNA‐Notch1 on the cell viability and Notch‐1 pathway proteins induced by Si‐MALAT1 was also studied. Furthermore, miR‐125b overexpressing MM cell lines were injected subcutaneously into nude mice. MiR‐125b and MALAT1 were inversely expressed in MM cell lines. Lenti‐Sh‐miR‐125b inhibited the expression of MALAT1 and Notch‐1 protein. Binding sites were confirmed between miR‐125b and MALAT1, and silencing MALAT1 did not alter the expression of Notch‐1. The apoptosis rate was increased and the survival rate was decreased obviously in GSI XII (targeted cleavage of Notch‐1 receptor) group, along with the inhibited Notch1 and HES1 proteins. Moreover, the decreased cell viability and Notch‐1 pathway proteins induced by Si‐MALAT1 could be reversed by pcDNA‐Notch1. Lenti‐Sh‐miR‐125b promoted survival and decreased Notch1 and HES1 proteins levels, while this effect was reversed by si ‐MALAT1. MiR‐125b regulated MALAT1 expression via Notch1 signaling pathway to regulate cell growth, thus participating in the occurrence and progression of MM, which functioned as a therapeutic target for tracking MM.

## Introduction

Multiple myeloma (MM) is a fatal hematological cancer with high mortality and morbidity 1, which is characterized by the clonal proliferation of malignant plasma cells, osteolytic bone destruction, and pathological fractures 2, 3. During the past decades, the incidence of MM has continued to rise, now it has become the second most common and life‐threatening neoplasm in USA 4. With the development of biochemistry science, various therapeutic advancements are continuously used to cope with MM, including autologous stem cell transplantation, application of proteasome inhibitors, and immune‐modulatory drugs 5. Although the availability of innovative drugs have improved clinical outlook, MM remains difficult to cure 6, the complex biological and core mechanisms have not yet been fully elucidated.

MicroRNAs (miRNAs) are small noncoding RNAs of 19‐25 nucleotides long, which negatively regulate mRNAs expression by inhibiting translation of target genes 7 and act as oncogenes or tumor suppressors 8. Emerging studies are screening miRNAs dysregulated in MM progression, for example, down‐regulation of miR‐15a and miR‐16 of primary MM cells contributes to the drug resistance and progression by the modulation of the bone marrow microenvironment 9. In addition, miR‐20a and miR‐17 are also participate in the tumorigenicity of MM 10. Circulating and serum‐derived miRNAs have also been identified in MM patients 11. Furthermore, dexamethasone‐induced miR‐125b induces cell death resistance mechanisms in MM cells via the p53/miR‐34a/SIRT1 signaling network 12. However, it remains largely unknown how miR‐125b regulates MM, and the identification of miR‐125b potential molecular mechanisms mediating MM are urgently needed.

Long noncoding RNAs (lncRNAs) are RNA transcripts with more than 200 nucleotides and without protein coding potential. Most of them function as master regulators and mediate the majority of vital pathways and processes including development, proliferation, differentiation, and serving as “miRNA sponges” 13. At present, various lncRNAs could act as biomarkers for predicting survival in MM patients 14. Metastasis‐associated lung adenocarcinoma transcript 1 (MALAT1) has been indicated to be overexpressed in MM patients and may serve as a molecular predictor of MM progression in early stage 15. In mesenchymal stem cells (MSCs), MALAT1 promotes the activation effect of the key transcription factor Sp1 on LTBP3 promoter by modulating recruitment of Sp1 to the LTBP3 gene that regulated the bioavailability of TGF‐*β* 16. Of note, MALAT1 is a predictable target of miR‐125b and miR‐125b suppresses bladder cancer development through down‐regulating the expression of MALAT1 17. This individual report highlights the urgent need to identify aberrant MALAT1 and to clarify their underlying mechanism in MM.

The Notch signaling pathway is one of the most commonly activated pathways in diverse cancers, which is involved in cell proliferation, differentiation, and survival 18. Recently, studies have indicated that Notch pathway is associated with the process of MM. Notch signaling dysregulation could promote MM‐associated bone disease 19. Activation of Notch pathway negatively regulated miR‐223 expression in MM‐BMMSCs, leading to increased VEGF and IL‐6 levels and reduced osteogenic differentiation, suggesting the important role of Notch signaling in MM 20. Nevertheless, it is not yet clear whether MALAT1 is involved in the regulation of MM through Notch signaling.

Here, we show that miR‐125b is down‐regulated, whereas MALAT1 and Notch1 are up‐regulated in MM cell lines. MALAT1 is identified as a target of miR‐125b and negatively regulated by miR‐125b. MALAT1 regulates MM cell proliferation through the Notch1 pathway. Overall, our findings demonstrate that miR‐125b is a tumor suppressor in MM, and provide a window that permits a better understanding of miR‐125b to be novel therapeutic targets.

## Material and Methods

### Informed consent

Informed consent was obtained from all individual participants and their families included in the study. Additional informed consent was obtained from all individual participants and their families for whom identifying information is included in this article. Moreover, the detailed information of the participants that were studied should not be published. This study was approved by the Research Ethics Committee of The First Affiliated Hospital of Inner Mongolia Medical University (Inner Mongolia, China).

### Cell culture and transfection

Peripheral blood mononuclear cells including PBMC1, PBMC2, PBMC3, and PBMC4, were donated by normal healthy individuals. Other eight MM cell lines (NCI‐H929, U266, SKMM1, MMIS, KMS‐12‐BM, MM1R, PRMI‐8226, and INA‐6) were purchased from American Type Culture Collection. All cell lines were maintained in RPMI‐1640 (Life Technologies, Carlsbad, CA) supplemented with 10% fetal bovine serum (Lonza Group Ltd., Switzerland) and 1% penicillin/streptomycin (Life Technologies) with 5% humidified CO_2_ at 37°C. The miR‐125b overexpression lentiviral vector (Lenti‐miR‐125b), miR‐125b down‐regulation lentiviral vector (Lenti‐Sh‐miR‐125b), MALAT1 Si‐RNA (Si‐MALAT1), and MALAT1 overexpression vector (pcDNA ‐MALAT1) were purchased from Qiagen (Hilden, Germany). After cultured in six‐well plates supplemented with antibiotic‐free medium, NCI‐H929 and PRMI‐8226 cells were transfected with lentiviral supernatant to obtain stable‐transfected cell lines and chosen for the following procedures.

### RNA extraction and quantitative real‐time PCR

Total RNA was extracted from cells by TRIzol reagent (Invitrogen, San Diego, CA) referring to the manufacturer's instructions and then stored at −80°C. The isolated RNA was reverse transcribed to obtain complementary DNA by PrimeScript RT reagent Kit (Invitrogen, Carlsbad, CA). The total obtained complementary DNA was operated to real‐time PCR using standard SYBR Green PCR kit (Applied Biosystems, Foster City, CA). All processes of PCR were performed in an ABI Prism 7500 system (Applied Biosystems). For carrying out the relative RNA expression, the 2^−△△CT^ method was performed. The expression levels of miR‐125b and MALAT1 were, respectively, normalized by U6 and GAPDH served as control.

### Western blot assay

After cells lysed in RIPA lysis buffer (Aidlab Biotechnology, Inc., China) with protease inhibitors (Roche) on ice, ultrasonication, and centrifugation, the whole protein was isolated. The protein was separated with 12% SDS‐PAGE and then transferred onto the polyvinylidene difluoride (PVDF) membrane (Millipore, Billerica, MA). The transferred membrane was maintained in antibodies against Bcl‐2, c‐caspase3, and *β*‐actin which served as loading control (Abcam, Cambridge, UK). Anti‐cleaved Notch1 (Val 1744) was purchased from Cell SignalingTechnology. After washing and incubating in HRP‐conjugated secondary antibodies, the protein transferred to membranes was defined with chemiluminescence detection kit (Aidlab Biotechnology) and quantified by Image Lab 4.0 software (Bio‐Rad Laboratories, Richmond, CA).

### MTT assay

For assessing the cell grown tendency and cell viability, the MTT assay was performed. These cells were planted in 96‐well plates to culture for corresponding times. Then 20 *μ*L MTT was added into each well to maintain it for 4 h at 37°C. After the medium was sucked and the cultivation was terminated, crystals were dissolved by adding 150 μL DMSO and agitating for 10 min at room temperature. The absorbance values in each well were surveyed using a microplate reader (Bio‐Rad Laboratories, Richmond, CA).

### Apoptosis detection

Apoptosis was measured with Annexin V‐PE/7AAD staining (Southern Biotech, Birmingham, AL) according to the manufacturer's instructions. After being washed twice with 37°C PBS and stained with Annexin V‐PE/7AAD, cells were analyzed by flow cytometry (FACScan, BD Biosciences), and apoptotic fractions were recorded with CELL Quest 3.0 software.

### Animals and in vivo of MM

BALB/c nude mice (5 weeks old) were provided by Beijing Vital River Laboratory Animal Technology Co. Ltd. (animal license number: SCXK (Beijing) 2012–0001). These animals were housed and monitored in our Animal Research Facility. Experimental procedures and protocols had been approved by Institutional Animal Care and Use Committee of the Affiliated Hospital of Inner Mongolia Medical University and conducted according to institutional guidelines. Animals were randomized into four groups (*n* = 5 in each group) and subcutaneously injected with NCI‐H929, PRMI‐8226 cells, NCI‐H929 transfected with lenti‐miR‐125b and PRMI‐8226 cells transfected with lenti‐miR‐125b, respectively. The tumor size was detected each day after transplantation. The digital caliper was used to examine the size of tumor including the length (L) and width (W). The tumor volumes were calculated following the formula: L*W^2^/2.

### Dual luciferase assay

The binding sites between MALAT1 and miR‐125b were predicted using DIANA tools (http://carolina.imis.athena-innovation.gr/diana_tools/web). Two fragments, which were shown in Figure 3, of MALAT1 containing the predicted the wild‐type (WT) and mutant (Mut) miR‐125b‐binding site in NCI‐H929 and PRMI‐8226 cells were chemically synthesized and cloned into the downstream of the luciferase gene of pmir GLO Dual Luciferase miRNA target expression vector between XhoI and NotI sites. For reporter assays, MM cells were cotransfected with wild‐type (mutant) reporter plasmid and miR‐Ribo^™^ mimics (miR‐Ribo^™^ negative control) using Lipofectamine 2000 (Invitrogen). Firefly and Renilla luciferase activities were measured in cell lysates using the Dual Luciferase Reporter Assay system. Luciferase activity was measured 48 h posttransfection using a Multimode Detector (Beckman Coulter, CA) according to the manufacturer's instructions (Promega, Madison, WI). Firefly luciferase units were normalized against Renilla luciferase units to control for transfection efficiency.

### RNA pull‐down assay

MALAT1 and its antisense RNA were in vitro transcribed and biotin‐labeled using a biotin RNA labeling mix (Roche, Indianapolis, IN) and T7/SP6 RNA polymerase (Roche), treated with RNase‐free DNase I (Roche) and purified using an RNeasy mini kit (Qiagen, Valencia, CA). One milligram of protein from NCI‐H929 and PRMI 8226 cell extracts was mixed with 50 pmol biotinylated RNA, incubated with streptavidin agarose beads (Invitrogen), and washed three times with NaCl/Pi at room temperature. The retrieved proteins were detected using a standard western blot technique.

### Statistical analysis

For the statistical analysis, SPSS 16.0 software was used to survey. All the data were indicated as means ± SD. A single comparison between two groups was surveyed by Student's *t* test, and the relationship between MALAT1 and miR‐125b expressions was tested with two‐tailed Pearson's correlation. *P <* *0.05 and **P <* *0.01 were considered statistically significant for the differences.

## Results

### Negative correlation existed between the expression of miR‐125b and MALAT1

**Figure 1:**
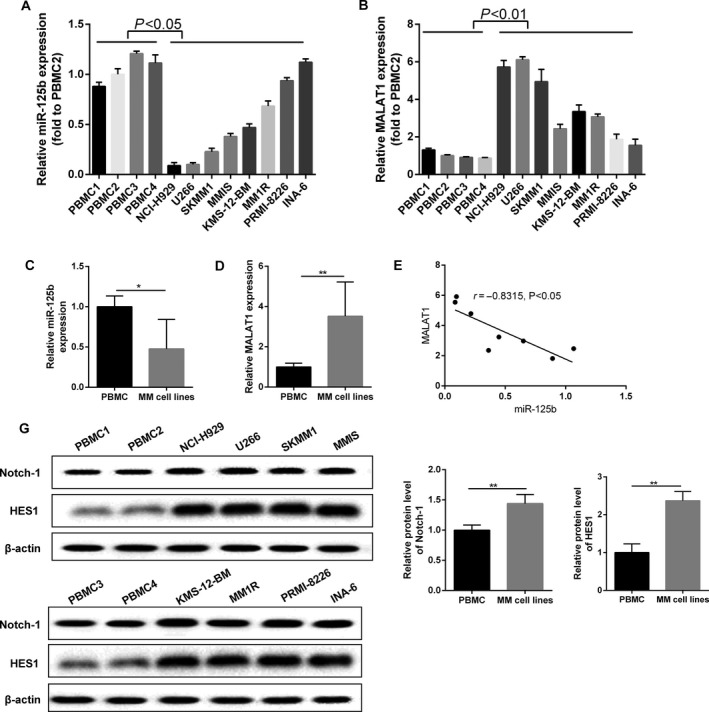
The relationship between miR‐125b and MALAT1 in MM cells. (A) The expression of miR‐125b was assessed using qRT‐PCR method in MM cell lines and normal peripheral blood mononuclear cells. (B) MALAT1 expression was assessed using qRT‐PCR method in MM cell lines and normal peripheral blood mononuclear cells. (C) The expression level of miR‐125b was down‐regulated in MM cell lines compared with PBMC cell lines. (D) MALAT1 was up‐regulated in MM cell lines compared with PBMC cell lines. (E) Negative correlation existed between the expression of miR‐125b and MALAT1. (F) Notch1 and HES1 protein was detected by western blot method. (G) Histograms of quantification protein concentration. Data are exhibited as mean ± SD. In qRT‐PCR, U6 and GAPDH served as controls, respectively. In western blot, *β*‐actin acted as internal reference. *P <* *0.05, **P <* *0.01 compared with controls.

In MM cell lines, including NCI‐H99, U266, SKMM1, MMIS, KMS‐12‐BM, and MM1R, the relative miR‐125b expression was significantly decreased when compared with the controls; while the gene expression of miR‐125b was relatively high in PRMI‐8226 and INA‐6 cells, nearly to the baseline (Fig. 1A). On the other hand, the situation of MALAT1 was contrary to those of miR‐125b. The expression of MALAT1 in NCI‐H99 and U266 cells reached maximum (Fig. 1B) compared to the control group. On the whole, the expression of miR‐125b in MM cell lines was lower (Fig. 1C) and the level of MALAT1 was higher (approximately 3.64‐fold) than peripheral blood mononuclear cells (Fig. 1D). Hence, as shown in Figure 1E, negative correlation existed between the expression of miR‐125b and MALAT1 (*r* = 0.8315, *P* < 0.05). Moreover, the protein expression of Notch1 and HES1 was up‐regulated in MM lines than PBMC cells (Fig. 1F), and the corresponding western blot histogram was presented in Figure 1G.

### The level of miR‐125b influenced Notch1 expression and MM cell growth

**Figure 2:**
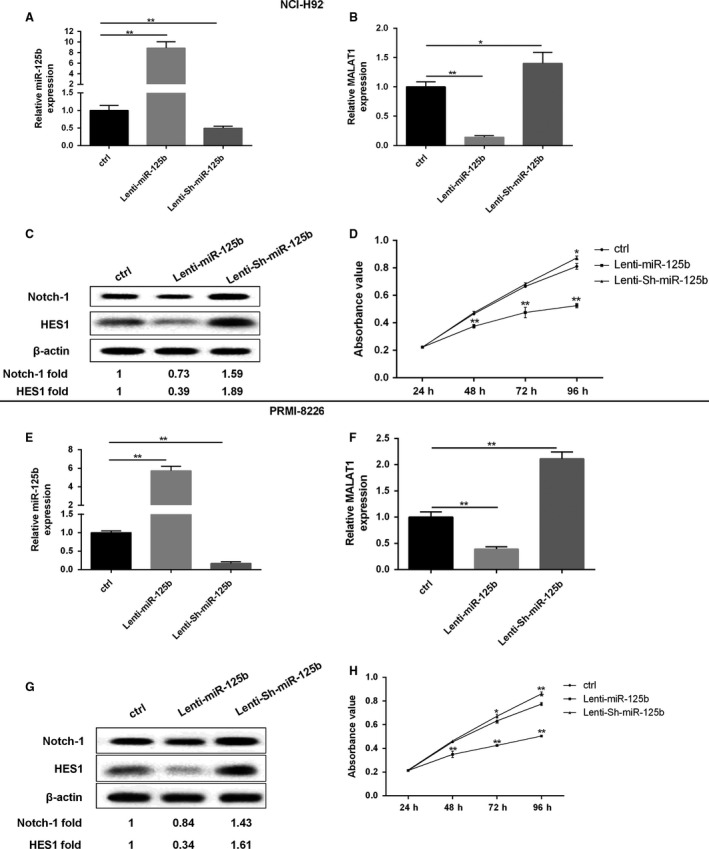
The expression of miR‐125b had an effect on MALAT1 and MM cell growth. In NCI‐H929 cells, (A, B) the expression of miR‐125b and MALAT1 was determined using qRT‐PCR method. (C) The protein level of Notch‐1 was assessed by western blot method. (D) The growth curves were examined by MTT assay. In PRMI‐8226 cells, (E, F) The expression of miR‐125b and MALAT1 was determined using qRT‐PCR. (G) The protein level of Notch‐1 and HES1 were assessed by western blot method. (H) The growth curves were examined by MTT assay. Data are exhibited as mean ± SD. U6 and GAPDH served as control in qRT‐PCR. *β*‐actin acted as internal reference in western blot. *P <* *0.05, **P <* *0.01 between groups comparison.

For further evaluating the role of miR‐125b in regulation of MALAT1, the expression of miR‐125b was detected. The overexpression (8.86‐fold to control) of miR‐125b in NCI‐H929 cells and the decline of MALAT1 expression that was only 0.17‐fold to control were observed when cells were transfected with Lenti‐miR‐125b; while the level of MALAT1 (1.4‐fold to control) was slightly enhanced in NCI‐H929 cells due to knockdown of miR‐125b (Fig. 2A and B). Similarly, the production of Notch1 and HES1 presented the same expression trend like MALAT1 (Fig. 2C). Compared with the control cells, the growth of NCI‐H929 cells transfected with Lenti‐miR‐125b was significantly inhibited. After culturing for 3 days, the amount of NCI‐H929 cells transfected with Lenti‐Sh‐miR‐125b was gradually higher than control (Fig. 2D). As the similar situation indicated in Figure 2E and F, the up‐regulation of miR‐125b (5.73‐fold to control) and the down‐regulation of MALAT1 (0.39‐fold to control) were observed in PRMI‐8226 cells transfected with Lenti‐miR‐125b, when compared with the control cells; while Lenti‐Sh‐miR‐125b transfection reversed the above effects. Meanwhile, a significant reduction in Notch1 and HES1 protein was observed in Lenti‐miR‐125b group (Fig. 2G). Lenti‐Sh‐miR‐125b treatment could enhance cell viability, while Lenti‐miR‐125b presented the opposite outcome (Fig. 2H).

### MiR‐125b mimic reduced the luciferase activities

**Figure 3:**
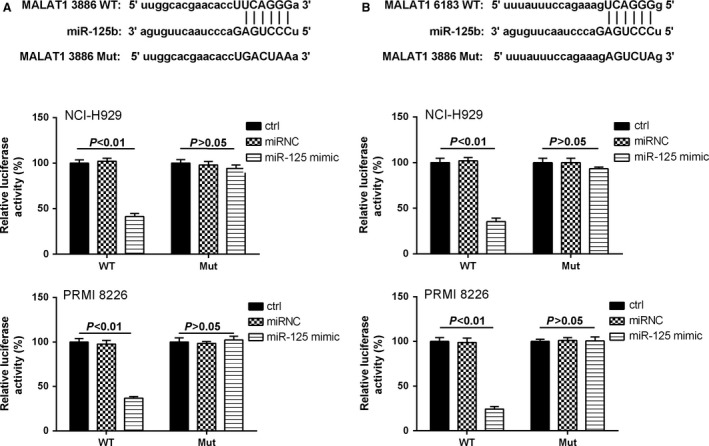
MiR‐125b mimic reduced the luciferase activities. (A) The first miR‐125b‐binding site predicted in MALAT1. The relative luciferase activities were inhibited in the wild‐type cells. (B) The second miR‐125b‐binding site predicted in MALAT1. The relative luciferase activities were inhibited in the wild‐type cells compared with mutate‐type cells. Data are exhibited as mean ± SD. *P *<* *0.01 compared with controls and miR‐125 mimic.

To explore the connection mechanism of miR‐125b and MALAT1, dual luciferase reporter assay was performed. Bioinformatics reveal that MALAT1 RNA contains two conserved target site of miR‐125b. Subsequently, the wild‐type sequence of MALAT1 (MALAT1‐WT) or its mutant sequence (MALAT1‐Mut) was subcloned into the pMIR luciferase reporter and then cotransfected with miR‐125b or miRNC into NCI‐H929 and PRMI 8226 cell lines. The relative luciferase activity of the pMIR‐MALAT1‐WT was significantly decreased in NCI‐H929 and PRMI 8226 cells cotransfected with miR‐125b mimic. However, the luciferase activity of pMIR‐ MALAT1‐Mut was unaffected in both MM cell lines by cotransfection with miR125b mimic (Fig. 3A and B). It suggested that both miR‐125b‐binding sites within MALAT1 were functional.

### MALAT1 regulated the Notch1 signaling pathway

**Figure 4:**
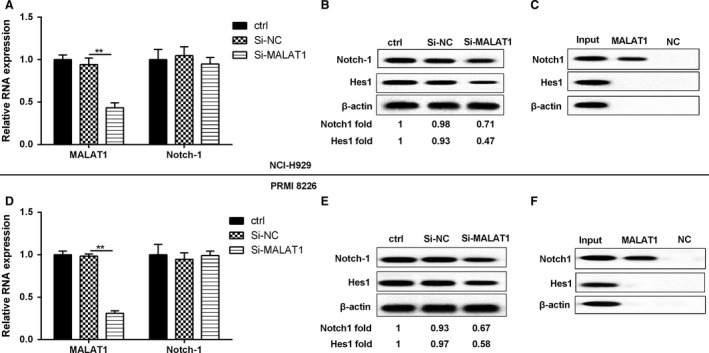
MALAT1 regulated the Notch1 pathway. NCI‐H929 and PRMI‐8226 MM cells were divided into three groups, namely controls, Si‐NC, and Si‐MALAT1. (A, D) The relative RNA expression of MALAT1 and Notch‐1 were assessed by qRT‐PCR. (B, E) The protein level of Notch‐1 and HES1 were detected by western blot. (C, F) RNA pull‐down was performed in order to confirm whether MALAT1 could bind Notch‐1. *β*‐actin acted as internal reference. **P <* *0.01 between groups comparison.

To further understand the regulation effect between MALAT1 and Notch1 signaling pathway, Si‐MALAT1 was transfected into NCI‐H929 and PRMI 8226 cells. From Figure 4A and D, the expression of MALAT1 was significantly decreased after silencing MALAT1, while the Notch‐1 mRNA was unchanged. In addition, the protein levels of Notch‐1 and HES1 were obviously decreased after NCI‐H929 or PRMI 8226 cell silencing MALAT1 (Fig. 4B and E). To further confirm the association between MALAT1 and Notch1, we performed an RNA pull‐down experiment. Figure 4C and F showed a significant enrichment of Notch1 (but not *β*‐actin protein enrichment) in the presence of MALAT1 RNA compared with antisense RNA (negative control). These results demonstrate a specific association between MALAT1 and Notch1.

### Notch1 signaling regulated MM cell growth

**Figure 5:**
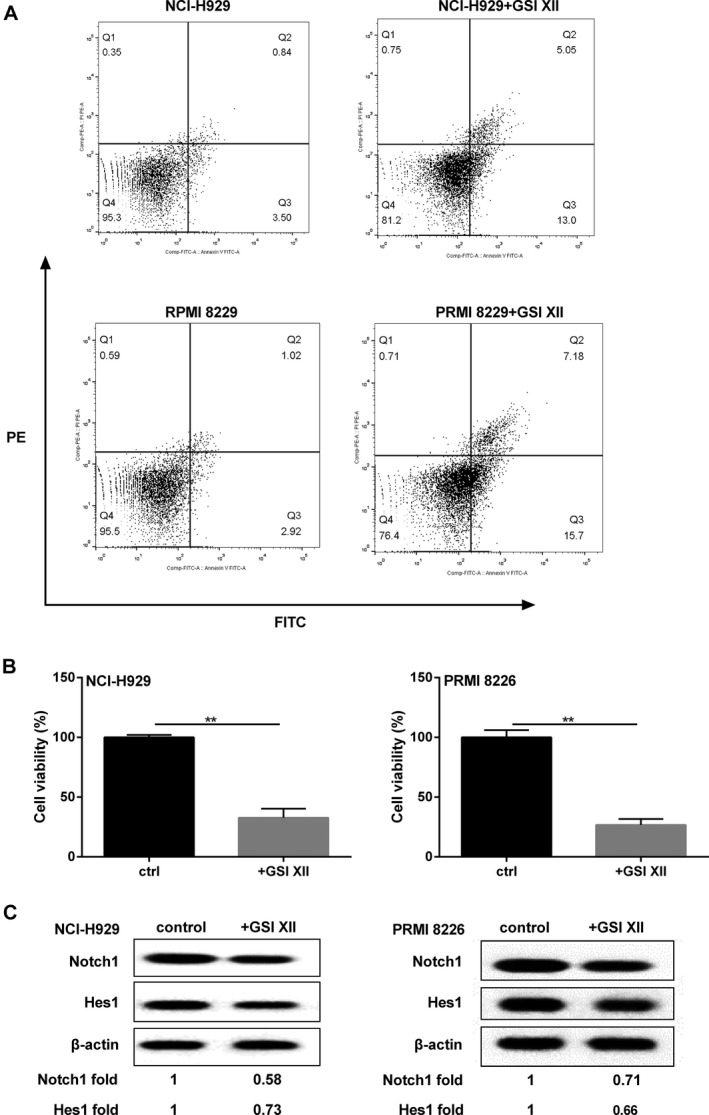
Notch1 signaling regulated MM cell growth. MM cells were incubated with 6 *μ*mol/L GSI XII, and detected the following parameters: (A) cell apoptotic rate. (B) cell survival rate. (C) Notch1 signaling pathway‐related proteins. *β*‐actin acted as internal reference. **P <* *0.01 between groups comparison.

The connection between Notch1 signaling and MM cell growth was worthy of in‐depth discussion. Based on cell culture, 6 *μ*mol/L GSI XII (targeted cleavage of Notch‐1 receptor) was incubated with cells. After incubation, the apoptosis rate was increased and the survival rate was decreased obviously in GSI XII group when compared to those of controls (Fig. 5A and B). Additionally, Notch1 signaling pathway was blocked, presenting the decreased Notch1 and HES1 protein level in GSI XII‐treated group of NCI‐H929 and PRMI8226 cells (Fig. 5C).

### MALAT1 regulated MM cell proliferation through the Notch1 signaling pathway

**Figure 6:**
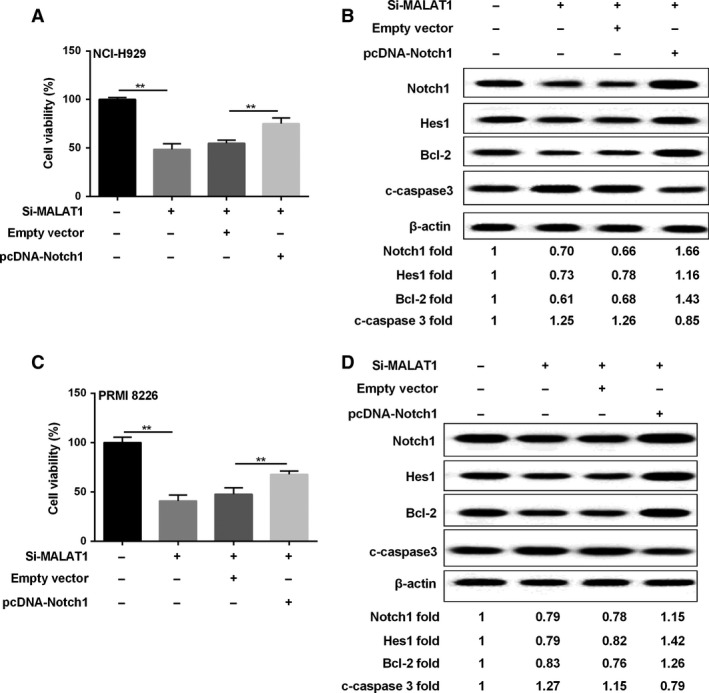
MALAT1 regulated MM cell proliferation through the Notch1 pathway. MM cells were divided into four groups: controls, Si‐MALAT1, empty vector, pcDNA‐Notch‐1. Then cell viability (A, C) and related proteins (B, D) were assessed by MTT and western blot method, respectively. *β*‐actin acted as internal reference. **P <* *0.01 between groups comparison.

We have known that MALAT1 regulated the Notch1 signaling pathway, and Notch1 signaling regulated MM cell growth, however, whether MALAT1 mediated MM cell proliferation via Notch1 pathway was unknown. After NCI‐H929 and PRMI8226 MM cells were treated with Si‐MALAT1, the cell growth was inhibited, while the effect could be reversed by transfection of pcDNA‐Notch‐1 (Fig. 6A and C). Furthermore, the protein expression of Notch1, HES1, and Bcl‐2 was reduced, and the protein level of c‐caspase3 was enhanced in MM cells silencing MALAT1, similarly, the regulation was strongly transformed after MM cells were transfected with pcDNA‐Notch‐1 (Fig. 6B and D).

### The expression of MALAT1 and miR‐125b played a key role in MM cell proliferation

**Figure 7:**
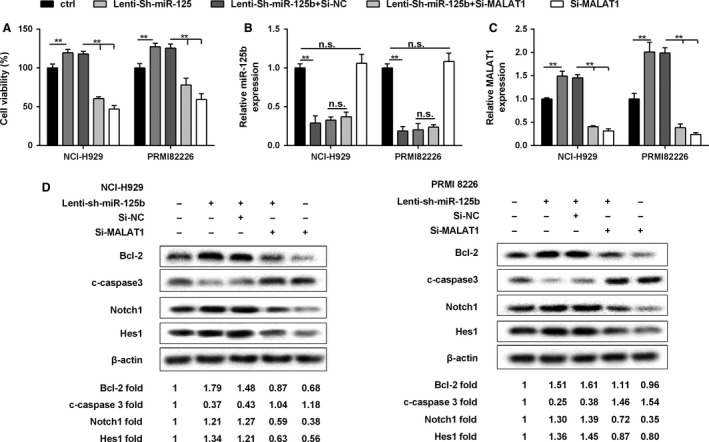
The expression of MALAT1 and miR‐125b played a key role in MM cell proliferation. NCI‐H929 and PRMI8226 cells were, respectively, transfected with Lenti‐Sh‐miR‐125b, Lenti‐Sh‐miR‐125b + Si‐NC, Lenti‐Sh‐miR‐125b + Si‐MALAT1, or Si‐MALAT1, (A) Cell viability was determined by MTT assay. (B, C) The expression of miR‐125b and MALAT1 was determined using qRT‐PCR. (D) The expression of Bcl‐2, c‐caspase3, Notch1, HES1, and *β*‐actin in MM cells by western blot. Data are exhibited as mean ± SD. U6, GAPDH, and *β*‐actin served as controls, respectively. **P <* *0.01 between groups comparison.

Compared with the MM cells infected with Lenti‐Sh‐miR‐125b, NCI‐H929 and PRMI‐8226 cells transfected with silencing MALA1 plus Lenti‐Sh‐miR‐125b showed decreased MM cell viability (Fig. 7A). As revealed in Figure 7B, the expression of miR‐125b was unchanged in cells transfected with Lenti‐Sh‐miR‐125b, Lenti‐Sh‐miR‐125b + Si‐NC, or Lenti‐Sh‐miR‐125b + Si‐MALAT1, while it was significantly decreased when compared with the control group. It showed that the expression of MALAT1 was promoted in cells transfected with Lenti‐Sh‐miR‐125b, but it was obviously inhibited by Si‐MALAT1 in NCI‐H929 and PRMI‐8226 cells with or without Lenti‐Sh‐miR‐125b (Figure 7C).

According to the western blot assay, both in NCI‐H929 and PRMI‐8226 cells, which had been transfected with Lenti‐Sh‐miR‐125b and si‐NC, the expression of Bcl‐2 was promoted and the regulation of c‐caspase3 was decreased compared to the control group; Conversely, the expression of Notch1 signaling pathway‐related proteins (Notch1, HES1) was increased. However, Si‐MALAT1 treatment could reverse the effects, showing decreased Bcl‐2, Notch1, and HES1, along with increased c‐caspase3 protein level (Fig. 7D).

### Overexpression of miR‐125b subcutaneously in mice inhibited tumor growth

**Figure 8:**
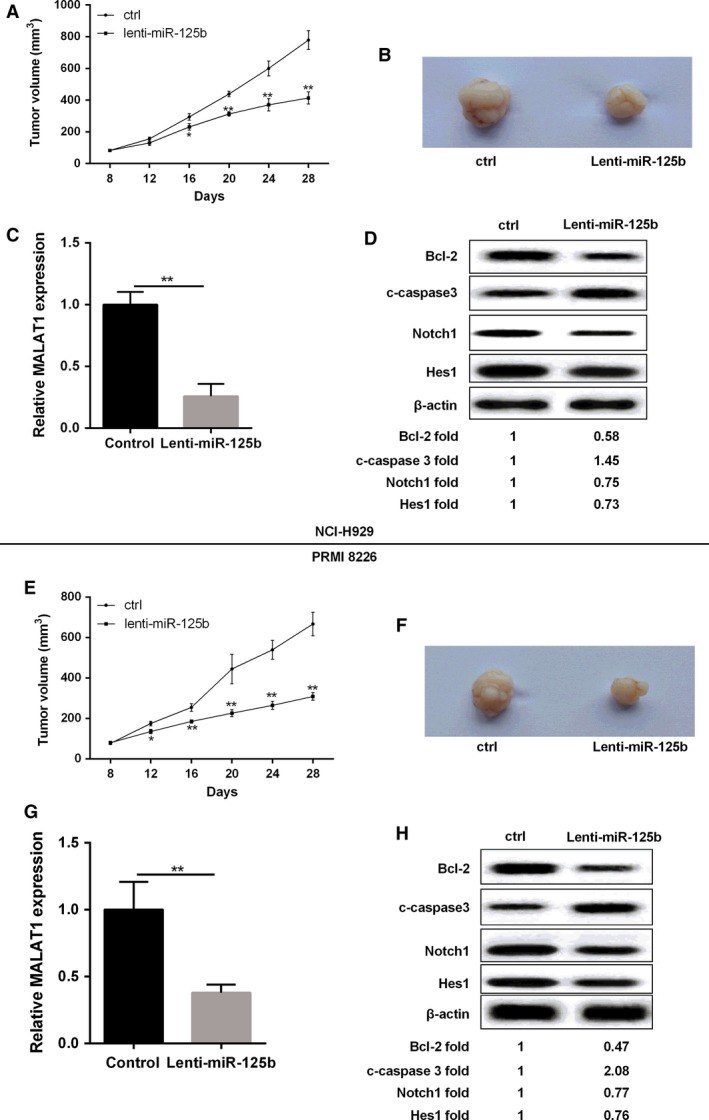
Over‐expression of miR‐125b inhibited tumor growth in nude mice. (A, E) NCI‐H929 or PRMI8226 cells transfected with lenti‐miR‐125b and control cells were subcutaneously injected into mice, and the tumor volume was detected. (B, F) Photographs of representative tumor formation in nude mice. (C, G) Relative MALAT1 expression in NCI‐H929 and PRMI8226 cells was detected by qRT‐PCR. (D, H) The expression levels of Bcl‐2, c‐caspase3, Notch1, HES1, and *β*‐actin in NCI‐929 or PRMI‐8226 cells transfected with lenti‐miR‐125b. Data are exhibited as mean ± SD. *β*‐actin served as the internal control. **P <* *0.01 compared with controls.

As shown in Figure 8A and E, NCI‐H929/PRMI‐8226 miR‐125b overexpressing cell lines were injected subcutaneously into mice, we found that compared with the control, the progression of tumor volume in subcutaneous MM cells was inhibited. The representative images about tumor growth were shown in Figure 8B and F. Lenti‐miR‐125b treatment obviously reduced the expression of MALAT1 in NCI‐H929 and PRMI 8226 cell lines (Fig. 8C and G). To further explore the expression of related proteins level, the western blot assay was performed in MM cells. The results illustrated that miR‐125b overexpression in MM cells could promote the expression of c‐caspase3 and depress the expression level of Bcl‐2, Notch1, and HES1 when compared to controls, and the protein expression results are listed below the western blot diagram (Fig. 8D and H).

## Discussion

Generally, miRNAs use two distinct mechanisms to regulate the expression of target genes. They are able to repress translation and/or decrease mRNAs when miRNAs and mRNAs are partially complementary, thus leading to rapid mRNA decay 21. In mammalian cells, miRNAs predominantly decrease target mRNA levels 22. For example, miR‐23b regulates targets at both mRNA and protein levels through partial sequence pairing with the target sites 23.

In this study, the expression levels of miR‐125b are down‐regulated in MM cell lines. Up‐regulation of miR‐125b could inhibit MM cells viability and suppress tumor growth, and increase cells apoptosis, while down‐regulation of miR‐125b has the reverse effects. These findings demonstrate that miR‐125b is a tumor suppressor in MM. Altogether, these findings prompt us to investigate the role of this miRNA in MM.

To date, a variety of oncogenic pathways have been identified as directly regulated by miR‐125b 24. Here, we perform a functional link between this miRNA and the oncogenic MALAT1 in MM. MALAT1 was an in silico predictable target of miR‐125b. The mature miR‐125b sequence was partially complementary with MALAT1 target sequences. Recent reports have indicated that MALAT1 directly binds to miR‐125b and regulates SIRT7 expression in bladder cancer 17; we investigated whether MALAT1 has similar inhibitory effects on miR‐125b levels in MM cells. The expression levels of MALAT1 were up‐regulated in MM cells. miR‐125b and MALAT1 were inversely expressed in MM cells. miR‐125b mimic down‐regulated, whereas miR‐125b inhibitor up‐regulated the expression of MALAT1. Binding sites were confirmed between miR‐125b and MALAT1. The effects of up‐regulation of miR‐125b were similar to that of silencing MALAT1 in MM cells. Taken together, these data indicated that MALAT1 is a true target of miR‐125b in MM cells. However, the exact mechanism remains unknown and warrants investigation.

Nowadays, diverse mechanisms are involved in the progression of MM, and Notch1 signaling pathway is one of them. Li et al. demonstrate that the specific *γ*‐secretase inhibitor (GSI) could effectively block Notch1 signal and inhibit MM cell growth and proliferation 25. Notch1 overexpression promotes MM cells growth and results in up‐regulation of VEGF expression, promotion of tumor growth, and increased microvessel density 26. Similarly, in this study, Notch1 signaling regulates MM cell growth and was regulated by MALAT1.

Finally, we demonstrated the in vivo anti‐tumor activity of miR‐125b mimics against human MM xenografts in Blab/c mice. To the best of our knowledge, this is the first evidence of a successful in vivo treatment with miR‐125b mimics in a murine xenograft model of human MM, which indeed has important potential toward clinical applications. Here, we illustrate that subcutaneous injection of miR‐125b mimics could result in significant tumor growth inhibition and decreased apoptosis suppressor gene Bcl‐2 expression, along with an induction of pro‐apoptotic gene c‐caspase3 level. These findings indicate that in vivo anti‐MM activity of miR‐125b mimics is related to the impairment of apoptosis signaling within MM xenografts. Further in vivo evaluation in preclinical models recapitulating the human BM milieu (huBMM), such as the SCID (severe combined immunodeficiency)‐hu model 27, will strengthen the translational value of miR‐125b mimics.

Taken together, our data suggest that loss of expression of miR‐125b contributes to the overexpression of MALAT1 through Notch‐1 pathway in MM. Therefore, the current findings not only provide new insight in clarifying the complex molecular mechanisms of specific miR‐125b and MALAT1 but also facilitate the development of miR‐125b and MALAT1‐directed therapeutic strategies for the disease.

## Conflict of Interest

None declared.
